# Rapid Acidification and Off-Flavor Reduction of Pea Protein by Fermentation with Lactic Acid Bacteria and Yeasts

**DOI:** 10.3390/foods13040588

**Published:** 2024-02-15

**Authors:** Dor Zipori, Jana Hollmann, Marina Rigling, Yanyan Zhang, Agnes Weiss, Herbert Schmidt

**Affiliations:** 1 Department of Food Microbiology and Hygiene, Institute of Food Science and Biotechnology, University of Hohenheim, Garbenstrasse 28, 70599 Stuttgart, Germany; dor.zipori@uni-hohenheim.de (D.Z.); jana.hollmann@uni-hohenheim.de (J.H.); 2 Department of Flavor Chemistry, Institute of Food Science and Biotechnology, University of Hohenheim, Fruwirthstrasse 12, 70599 Stuttgart, Germany; marina.rigling@uni-hohenheim.de (M.R.); yanyan.zhang@uni-hohenheim.de (Y.Z.); 3 Food Microbiology, Hamburg School of Food Science, University of Hamburg, Ohnhorstsrasse 18, 22609 Hamburg, Germany; agnes.weiss@uni-hamburg.de

**Keywords:** pea protein, fermentation, co-cultures, lactic acid bacteria, yeast, hexanal, *Yarrowia lipolytica*

## Abstract

Pea protein is widely used as an alternative protein source in plant-based products. In the current study, we fermented pea protein to reduce off-flavor compounds, such as hexanal, and to produce a suitable fermentate for further processing. Laboratory fermentations using 5% (*w*/***v***) pea protein suspension were carried out using four selected lactic acid bacteria (LAB) strains, investigating their growth and acidification capabilities in pea protein. Rapid acidification of pea protein was achieved with *Lactococcus lactis* subsp. *lactis* strain LTH 7123. Next, this strain was co-inoculated together with either the yeasts *Kluyveromyces lactis* LTH 7165, *Yarrowia lipolytica* LTH 6056, or *Kluyveromyces marxianus* LTH 6039. Fermentation products of the mixed starter cultures and of the single strains were further analyzed by gas chromatography coupled with mass spectrometry to quantify selected volatile flavor compounds. Fermentation with *L. lactis* LTH 7123 led to an increase in compounds associated with the “beany” off-flavors of peas, including hexanal. However, significant reduction in those compounds was achieved after fermentation with *Y. lipolytica* LTH 6056 with or without *L. lactis* LTH 7123. Thus, fermentation using co-cultures of LAB and yeasts strains could prove to be a valuable method for enhancing quality attributes of pea protein-based products.

## 1. Introduction

The global demand for animal-based products has increased significantly in recent years, primarily based on the world’s continuous population growth and the adaptation of a Western-style diet rich in dairy and meat products throughout the entire world [1,2]. With the global population expected to reach 10 billion in 2050, traditional food production will be unable to meet global food requirements [3]. Simultaneously, consumers in developed countries are increasingly seeking plant-based alternatives to animal-based products, primarily for environmental, health, and animal welfare reasons [4]. As a result, the food industry is under pressure to develop plant-based products, which can also be a solution to the world’s future food demand.

Because legumes contain high levels of protein (18–32%), dietary fibers, minerals, and vitamins that are necessary for human health, as well as being cost-effective, they can be considered an adequate alternative protein source for the production of meat and dairy alternatives [5]. Currently, soy is the most commonly used plant protein substitute for animal proteins [6]. Other legumes, particularly peas, are gaining popularity as alternative protein sources with lower allergenicity than soy [7]. The pea may have a future as a sustainable human food supply due to its high yields, wide availability, and low production costs [8,9]. Peas (*Pisum sativum*) have a high protein content (20–25%), as well as fat (1.0–2.0%), carbohydrates (24–49%) (mostly starch), and dietary fiber (60–65%) [10]. They are also a good source of water-soluble vitamins, particularly those from the B-group [11,12]. Pea protein contains the essential amino acids lysine, histidine, and threonine in higher amounts than in other plant proteins, but is poor in methionine and tryptophan as well as cysteine [13,14,15].

However, there are several disadvantages associated with the adoption of pea proteins as a substitute for animal proteins. Plant proteins often exhibit undesirable characteristics such as poor color, lower solubility, and reduced contribution to important technical processes such as foam formation, emulsification, and gelation, when compared to proteins found in milk and meat [16]. Peas also contain antinutritional factors such as phytic acid and trypsin inhibitors [17,18,19]. Nonetheless, the presence of off-flavors described as “beany” or “green and grassy” is the main factor influencing consumer acceptance of pea protein-based products [7]. These unpleasant flavors are either naturally present in peas or are generated during the production process [20,21]. Hexanal, a product of the degradation of linoleic and linolenic acid, has been identified as a significant contributor to the off-odors associated with peas [21]. 1-Octen-3-ol, nonanal, 2-butyl-3-methoxypyrazine, 2-pentylfuran, (*E*,*E*)-2,4-decadienal, and 1-hexanol are other volatile compounds involved in the formation of “beany” and “green” off-flavors as well as many other aldehydes, alcohols, furans, and pyrazines [22,23,24]. In order to tackle these challenges, extensive research is currently underway to enhance the understanding of the characteristics of pea proteins and explore novel methods for their modification [10,25]. Among these approaches, the utilization of microbial fermentation stands out as a leading and promising strategy to enhance the sensory, functional, and nutritional attributes of pea proteins [26,27,28,29].

In recent years, numerous approaches have been tested for the fermentation of pea proteins. One common method involves utilizing a pea protein suspension in water or creating an emulsion by incorporating vegetable oil, which serves as the fermentation matrix for the process [27,28,29]. Lactic acid bacteria (LAB) have been widely employed as starter cultures for the fermentation of pea proteins due to their versatility and established safety record [30]. The fermentation of pea-protein-based products, similar to most fermented dairy or meat products, leads to a pH drop due to the production of lactic acid by LAB [30]. Consequently, the structure of the pea protein network undergoes changes, which, depending on protein concentration, can result in gel formation [31,32]. Furthermore, the resulting low pH-values may prevent the growth of spoilage microorganisms and can also inhibit bacterial endospore germination. Strains capable of growing in the pea protein matrix have been either isolated through screening processes [29] or obtained from commercially available starter cultures suitable for plant-based raw materials [27,28]. In a screening experiment conducted by Ben-Harb et al. (2019), which involved 55 strains isolated from cheese and vegetable products, only eight strains from the Actinobacteria and Proteobacteria phyla could grow individually or in combination within a pea protein emulsion. Nonetheless, the majority of the tested Firmicutes, yeasts, and molds exhibited growth either alone or in combination with other strains. Notably, among the Firmicutes species capable of growing in the emulsion were LAB species such as *Lactiplantibacillus plantarum*, *Lacticaseibacillus rhamnosus*, and *Lactococcus lactis*, as well as yeasts like *Yarrowia lipolytica*, *Kluyveromyces marxianus*, and *Geotrichum candidum* [29]. Furthermore, it has been demonstrated that fermentation with microbial consortia consisting of various strains can positively impact the aroma of the final product.

Schindler et al. (2012) demonstrated that lactic acid fermentation with *Pediococcus pentosaceus* and *L. plantarum* could lead to a reduction in hexanal levels [26]. In another study, fermentation with *L. plantarum* was shown to enhance the sensory properties of pea proteins while also lowering the pH from 6.5 to 4.6. However, this fermentation process resulted in decreased solubility and emulsifying capacity of the pea proteins [7].

El Youssef et al. (2020) conducted a study in which pea protein suspensions supplemented with sucrose were fermented using co-cultures of LAB and yeasts obtained from commercially available starter cultures. The co-cultures consisted of a consortium comprising *Lactobacillus acidophilus*, *Streptococcus thermophilus*, *Lactobacillus delbrueckii* subsp. *bulgaricus*, and one of three yeasts: *Torulaspora delbrueckii*, *Kluyveromyces lactis*, or *K. marxianus*. Aroma analysis of the fermentation products revealed that many off-flavor components, particularly aldehydes, ketones, and furans, were completely eliminated. Although the yeasts produced ethanol at concentrations ranging from 1.7 to 4.8 g/L, they also generated a variety of esters with fruity odors, which masked the off-flavors in the final products [28]. The findings suggest that the selection of starter cultures plays a crucial role in influencing various properties of the final product. It is evident that the use of multiple microorganisms in a co-culture as a starter culture can have a more substantial impact than employing a single strain alone. This highlights the importance of considering the synergistic effects and interactions among different microorganisms when designing fermentation processes for pea protein-based products.

The aim of the current study was the characterization of mixed starter cultures consisting of fast-acidifying LAB strains, as well as yeast culture specifically chosen for its ability to remove off-flavors, with particular emphasis on addressing hexanal as the primary component.

## 2. Materials and Methods

### 2.1. Pea Protein

A commercial pea protein isolate of *Pisum sativum* L. (Pisane F9*, Lot-Nr. 2021137414) was obtained from the company Cosucra (Warcoing, Belgium). According to the manufacturer’s specifications, the product had a dry matter content of 96.0% and contained 86.5% protein and 9% fat. Pisane F9* was kept under ambient conditions at room temperature, away from direct light exposure.

### 2.2. Materials and Chemicals

Glucose monohydrate was purchased from Merck KGaA (Darmstadt, Germany). Decanal (96%), heptanal (97%), 1-octanol (99%), 1-octen-3-ol (98%), and 2-pentylfuran (98%) were purchased from Alfa Aesar (Karlsruhe, Germany). (*E*,*E*)-2,4-Decadienal (95%) and 2-isobutyl-3-methoxypyrazine (99%) were purchased from J&K Scientific GmbH (Pforzheim, Germany). Hexanal (99%), 1-hexanol (99%), nonanal (95%), 2-nonanone (99%), and (*E*)-2-octenal (95%) were purchased from Sigma-Aldrich (Darmstadt, Germany). Ethanol (100%) was purchased from VWR Chemicals (Bruchsal, Germany). Ethyl hexanoate (99%), isoamyl acetate (99%), and 2-methylfuran (99%) were purchased from Acros Organics (Geel, Belgium).

### 2.3. Microbial Strains

The microbial strains used in this study are listed in Table 1. All strains were obtained from the strain collection of the Department of Food Microbiology and Hygiene, University of Hohenheim (Stuttgart, Germany).

### 2.4. Cultivation of Microbial Strains and Inoculum Preparation

*Lactococcus* and *Streptococcus* strains were routinely grown in M17 broth and on M17 agar. All other LAB strains were grown in Man Rogosa and Sharpe (MRS) agar and broth (all Merck KGaA, Darmstadt, Germany). Overnight cultures were prepared by inoculating 2–3 colonies of one strain in 10 mL of the respective broth and incubating them at 30 °C or 37 °C in the case of *S. thermophilus*, *L. rhamnosus*, and *L. bulgaricus* under a modified gas atmosphere (10% air, 10% carbon dioxide, 80% nitrogen). Yeast strains were cultivated on yeast extract, peptone, dextrose (YPD) agar, containing 1% yeast extract, 2% peptone from meat, 2% glucose, 1.5% agar-agar, and incubated at 25 °C under aerobic conditions for 48 h. Overnight cultures were prepared by inoculating one yeast colony in 50 mL YPD broth and incubating at 25 °C while shaking at 180 rpm. Cells were harvested after overnight incubation by centrifuging a 10 mL overnight culture at 4200 rpm (3400× *g*) at 4 °C for 15 min. The cell pellet was washed and resuspended in sterile 0.9% sodium chloride solution, then serially diluted to obtain 1 mL inoculum with 10^7^ or 10^6^ CFU/mL of LAB and yeast strains, respectively.

### 2.5. Preparation of Fermentation Mixture

A pea protein suspension was prepared by mixing 52.6 g of pea protein isolate powder with 1000 mL deionized water. The mixture was stirred using a magnetic stirrer at room temperature until a homogeneous suspension was obtained. The suspension was then autoclaved at 105 °C for 30 min. This process was required to eliminate the autochthonous microbiota of pea protein isolates prior to fermentation, according to Engels et al. (2022) [27]. Following cooling to ambient temperature, a sterile 25% (*w*/*v*) glucose solution was added and homogenously blended with the suspension, resulting in a 5% (*w*/*v*) pea protein isolate and a 1% (*w*/*v*) glucose suspension.

### 2.6. Development of a Rapid Acidification Test of Pea Protein

A rapid acidification test of pea protein by homofermentative LAB strains was performed in 24-well plates (Greiner CELLSTAR^®^ plates, 2 mL, Merck KGaA, Darmstadt, Germany) with a total working volume of 1.5 mL pea protein suspension (5% (*w*/*v*) pea protein isolate, 1% (*w*/*v*) glucose) after addition of 15 µL of the inoculum. The bacterial strains were introduced into the wells to ensure a uniform starting cell density of 10^5^ CFU/mL in every single well. This inoculation was performed in duplicate, with two wells allocated per strain. Negative controls were prepared by the addition of sterile 0.9% (*w*/*v*) sodium chloride solution instead of the inoculum. The plates were incubated aerobically, without shaking, at 30 °C for 16 h. After 16 h, 60 µL of 200 mg/L methyl red solution (Merck KGaA, Darmstadt, Germany) was added into each well. The observed color changes were documented and then compared against the color of a methyl red pH scale. This scale was generated by adjusting the pH of pea protein with a 1 M HCl solution using a pH meter (Testo 206-pH2, Testo SE & Co. KGaA, Titisee-Neustadt, Germany).

### 2.7. Batch Fermentation

Fermentation experiments were carried out in glass jars with a volume of 250 mL, containing 100 mL fermentation substrate of 5% (*w*/*v*) pea protein isolate, 1% (*w*/*v*) glucose. The volume of the inoculum was adjusted to 1 mL cell suspension with the appropriate cell density. In the case of single-strain fermentation, the starting cell density of LAB strains was set to 10^5^ CFU/mL, and that of the yeast strains to 10^4^ CFU/mL, while in mixed-strain fermentation the cell density was adjusted to 10^5^ and 10^4^ CFU/mL of LAB and yeast strains, respectively. Uninoculated control samples contained 1 mL 0.9% (*w*/*v*) sterile sodium chloride solution instead of the inoculum.

Inoculated fermentation mixtures were incubated aerobically, without shaking, at 30 °C in an incubator. The fermentation duration of pea protein with single LAB strains was limited to 24 h, while for yeast and mixed strain fermentation for 48 h. Directly after inoculation, after 16 h (LAB only), after 24 h, and after 48 h the pH was measured using a pH meter (Testo 206-pH2, Germany). Viable counts of the LAB strains were determined by surface plating in technical duplicates on M17 or MRS agar after two days of incubation at 30 °C under a modified gas atmosphere (10% air, 10% carbon dioxide, 80% nitrogen). Viable counts of the yeast strains were determined by surface plating in duplicate using yeast–glucose–chloramphenicol agar (YGC agar, Merck KGaA, Darmstadt, Germany) after three days of incubation at 25 °C. In the case of mixed-strain fermentation, M17 agar was supplemented with a final concentration of 50 mg/L of amphotericin B (Sigma-Aldrich, Darmstadt, Germany). The fermentation products were stored at −20 °C for further analysis. Single-strain LAB fermentation experiments were performed in two biological replicates. All other fermentations were carried out in triplicate.

### 2.8. Biochemical Analysis

The concentrations of L-lactic acid and ethanol were determined using commercial enzymatic UV assays (both R-Biopharm AG, Pfungstadt, Germany). After thawing, the samples were diluted and heated to 95 °C for 15 min and later centrifuged at 12,000× *g* for 15 min. The supernatants were cooled to room temperature and either analyzed directly or stored at −20 °C until use. Both enzymatic assays were measured at a wavelength of 340 nm according to the manufacturer’s instructions.

The free amino acid concentration and composition was determined at the Core Facility Hohenheim (CFH) using the method for determination of amino acids except tryptophan in accordance with EU regulation EC No 152/2009 Annex III—F-G [33]. Amino acids were determined after previous extraction with sodium citrate loading buffer (pH 2.2) or mild hydrochloric acid (0.1 mol HCl/L) of the sample matrix and eventually the co-extracted macromolecules containing nitrogen were treated with sulfosalicylic acid and eliminated by filtration, then adjusted to pH 2.2. The free amino acids were separated by ion exchange chromatography and determined photometrically at 570 nm after ninhydrin post-column derivatization. For the determination of total tryptophan, the sample was hydrolyzed under alkaline conditions with saturated barium hydroxide solution and heated to 110 °C for 20 h. Subsequently, free tryptophan was extracted under mild acidic conditions. The determination of total and free tryptophan was carried out using high-performance liquid chromatography (HPLC) with fluorescence detection (Agilent series 1200, Agilent Technologies Inc., Waldbronn, Germany)

### 2.9. Instrumental Aroma Analysis by HS-SPME-GC-MS

To investigate the aroma profile of the non-fermented and fermented pea protein samples, gas chromatography-mass spectrometry (GC-MS) analysis was carried out. A total of seven fermentation products were analyzed for the presence or absence of selected aroma compounds after 48 h of fermentation together with the inoculated control sample. In addition to those samples, an acidified pea protein sample was tested, in order to examine a possible effect of the pH-value on the detection of volatile compounds in pea protein. This sample was prepared by acidifying the pea protein suspension to a pH-value of 4.5 using a 4 M HCl stock solution after 24 h of incubation at 30 °C. Subsequently, a further adjustment was made to achieve a pH of 4.3 after a total incubation period of 48 h. A total of 15 target aroma compounds (Table 2) were selected from the literature and identified in the samples via authentic standard solutions.

The extraction of the aroma components from the samples was performed by headspace-solid phase microextraction (HS-SPME) coupled with a multipurpose sampler (MPS) autosampler (Gerstel, Muelheim an der Ruhr Germany) with a CAR/PDMS fiber (carboxene/polydimethylsiloxane, 85 µm, 1 cm length, Supelco, Darmstadt, Germany). Prior to aroma analysis, the samples were agitated for 10 min at 45 °C and 250 rpm, followed by headspace extraction at the same temperature for 15 min. Afterwards, the analytes were directly desorbed in the split/splitless inlet at 150 °C (split 1:2) using a SPME liner of a gas chromatography equipped with a mass spectrometry detector for 1 min. The analytes were transferred to the gas chromatograph (Agilent Technologies, Waldbronn, Germany) equipped with a DB-WAX UI column (30 m × 0.25 mm × 0.25 µm; Agilent Technologies, Santa Clara, CA, USA). Helium (5.0, Westfalen, Muenster, Germany) with a constant flow rate of 1.2 mL/min was used as carrier gas. As a starting temperature, the GC oven was held at 40 °C for 3 min, then increased to 220 °C with a rate of 5 °C/min and maintained for 10 min. Molecules were ionized at an ionization energy of 70 eV with an ionization source temperature of 230 °C and a quadrupole temperature of 150 °C.

The mass spectrum was recorded in scan mode (*m*/*z* 33–350), and the solvent delay was 1.5 min. The measurement was performed in technical triplicate from two biological replicates (*n* = 6). Target compounds were identified by retention index, NIST mass spectra database, and comparison to authentic standards. This evaluation of the GC-MS data was performed using two types of software: MassHunter Qualitative Analysis (version B.08.00) and MassHunter Quantitative Analysis (version B.08.00). The amount of each aroma compound was determined based on a calibration of the peak area of external standards with known concentrations. The odor activity value (OAV), indicating the contribution of an aroma compound to the overall aroma, was calculated using the determined concentrations of the aroma compounds in the fermentation products divided by their respective odor thresholds in water, reported in the literature.

Table 2 lists the aroma compounds and their odor descriptors along with their odor threshold in water.

### 2.10. Sensory Analysis

The odor impressions of the non-fermented and fermented samples were evaluated by five experienced assessors (3 females, 2 males, all non-smokers, mean age 28 years) from the University of Hohenheim (Stuttgart, Germany) [42,43,44]. All assessors had participated in sensory training for at least 1 week, which was held at the Department of Flavor Chemistry, University of Hohenheim. The samples from the two biological replicates underwent a simple descriptive analysis without given references by the five assessors. The results from the individual panelists were subsequently collected and summarized.

### 2.11. Statistical Analysis

The statistical analysis was conducted using the software OriginPro 2023 (OriginLab Corporation, Northampton, MA, USA). To assess the normality of the data distribution, the Wilk–Shapiro test was employed. Outliners in the quantification of the GC-MS analysis were identified using Grubbs’s test and were not taken into consideration. One-way analysis of variance (ANOVA) was performed for data comparison. When more than two groups were tested, mean values were compared using Tukey’s post hoc test. All tests were performed at *p* = 0.05, except the Grubbs’s test which was performed at *p* = 0.1.

## 3. Results

### 3.1. Qualitative Assessment of the Acidification Capability of Homofermentative LAB in Pea Protein

**Figure 1:**
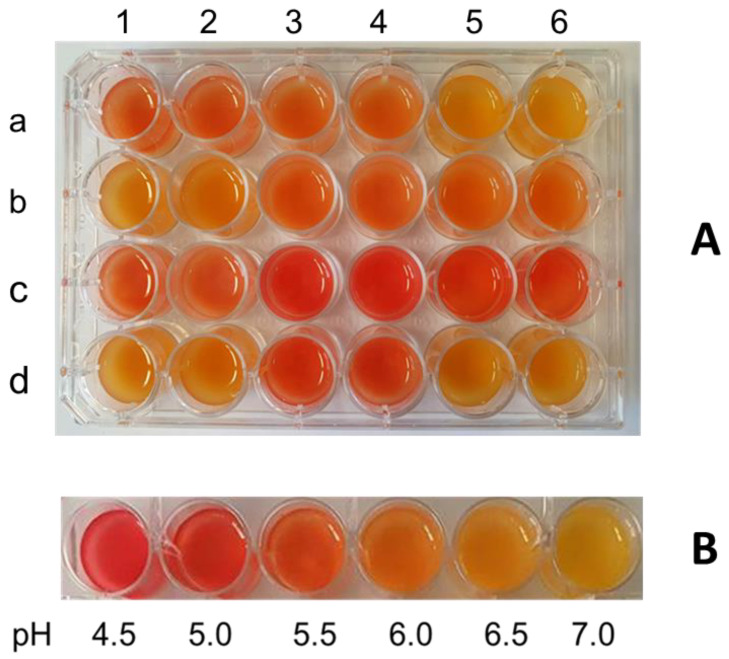
Acidification test with methyl red in a 5% (*w*/*v*) pea protein suspension after 16 h of fermentation with homofermentative LAB strains. (**A**) a1,a2: *L. curvatus* LTH 955; a3,a4: *L. rhamnosus* LTH 1409; a5,a6: *P. acidilactici* LTH 1412; b1,b2: *L. sakei* subsp. *sakei* LTH 3745; b3,b4: *L. plantarum* LTH 6724; b5,b6: *P. pentosaceus* LTH 6727; c1,c2: *L. cremoris* LTH 7122; c3,c4: *L. lactis* subsp. *lactis* LTH 7123; c5,c6: *S. thermophilus* LTH 7138; d1,d2: *L. delbrueckii* subsp. *bulgaricus* LTH 7139; d3,d4: *L. lactis* subsp. *lactis* LTH 7163; d5,d6: uninoculated control. (**B**) pH color scale in pea protein from 4.5 to 7.0.

In order to analyze bacterial strains for rapid acidification of the fermentation substrate, a rapid acidification test using a pea protein suspension inoculated with LAB was established with 11 selected LAB strains. The pH indicator dye methyl red was mixed with the samples of the pea protein suspension after 16 h of fermentation to allow visual assessment of the acidification capabilities of the LAB strains. Figure 1A illustrates the color changes with methyl red after a 16 h fermentation with LAB, together with a pH color scale (Figure 1B) of methyl red in pea protein suspension.

The most intensive color changes from yellow (neutral) to red (acidic) were observed for *L. lactis* subsp. *lactis* LTH 7123 (Figure 1A, wells c3–c4), *S. thermophilus* LTH 7138 (Figure 1A, wells c5–c6), and *L. lactis* subsp. *lactis* LTH 7163 (Figure 1A, wells d3–d4). The full red color of these samples was comparable in intensity to the pH 4.5 standard (Figure 1B), indicating a strong acidification up to a pH of 4.5. *L. curvatus* LTH 955 (Figure 1A wells a3–a4), *L. rhamnosus* LTH 1409 (Figure 1A, wells a5–a6), *L. plantarum* LTH 6724 (Figure 1A, wells b3–b4), *L. cremoris* LTH 7122 (Figure 1A, wells c1–c2), and *P. pentosaceus* LTH 6727 (Figure 1A, wells b5–b6) displayed a moderate acidification to pH-values of between pH 5.5 and 6.0. *P. acidilactici* LTH 1412 (Figure 1A, wells a5–a6), *L. sakei* subsp. *sakei* LTH 3745 (Figure 1A, wells b1–b2), and *L. delbrueckii* subsp. *bulgaricus* LTH 7139 (Figure 1A, wells d1–d2) showed only a weak acidification in the range of 6.5 to 6.0. While the test delivered pH-values as a range instead of precise values, it remains highly beneficial for differentiating between fast and strong acidifiers and slower and weaker ones.

### 3.2. Rapid Acidification of Pea Protein by L. lactis LTH 7123

**Figure 2:**
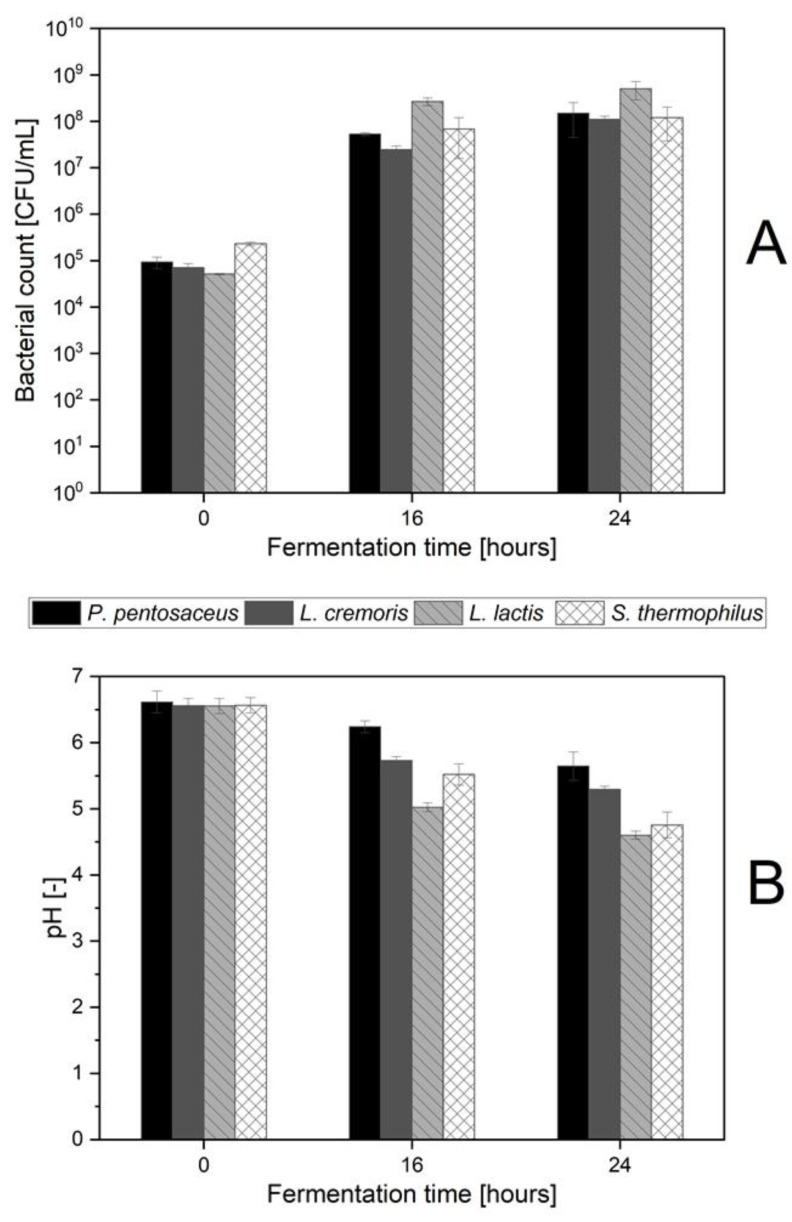
Changes in viable counts and pH-values during fermentation of pea protein suspension with different homofermentative LAB. *P. pentosaceus* LTH 6727, *L. cremoris* LTH 7122, *L. lactis* LTH 7123, *S. thermophilus* LTH 7138. (**A**) Viable cell counts (CFU/mL) of the selected strains throughout the fermentation. (**B**) pH-values of pea protein during 24 h of fermentation.

To obtain more precise data, scaled-up fermentation experiments were conducted using suspensions composed of 5% (*w*/*v*) pea protein isolate and 1% (*w*/*v*) glucose. Four of the strains employed in the rapid test described above were used to investigate their growth behavior and acidification capabilities: *P. pentosaceus* LTH 6727, *L*. *cremoris* LTH 7122, *L. lactis* subsp. *lactis* (from here simply *L. lactis* LTH 7123), and *S. thermophilus* LTH 7138. This experiment aimed to validate the efficacy of the rapid test and explore the correlation between pH reduction and microbial growth. The results are depicted in Figure 2A,B.

These results confirm that the *L. lactis* LTH 7123 acidified the pea protein matrix more rapidly and strongly than any other of the used strains (Figure 2B). It reduced the pH of the suspension from an initial value of 6.5 to a pH-value of 5.0 after 16 h and 4.6 after 24 h. In comparison, the second fastest acidifier *S. thermophilus* LTH 7138 reached only a pH of 5.5 after 16 h. Among the four strains, *P. pentosaceus* LTH 6727 caused the lowest pH reduction. This finding is consistent with the results of the rapid acidification test. In terms of growth *L. lactis* LTH 7123 also achieved the highest counts after 16 and 24 h of fermentation (Figure 2A), with cell densities of 2.7 × 10^8^ CFU/mL and 5.2 × 10^8^ CFU/mL, respectively. *L. lactis* LTH 7123 was therefore selected for the mixed strain fermentation.

### 3.3. Fermentation of Pea Protein with Mixed Cultures

**Figure 3:**
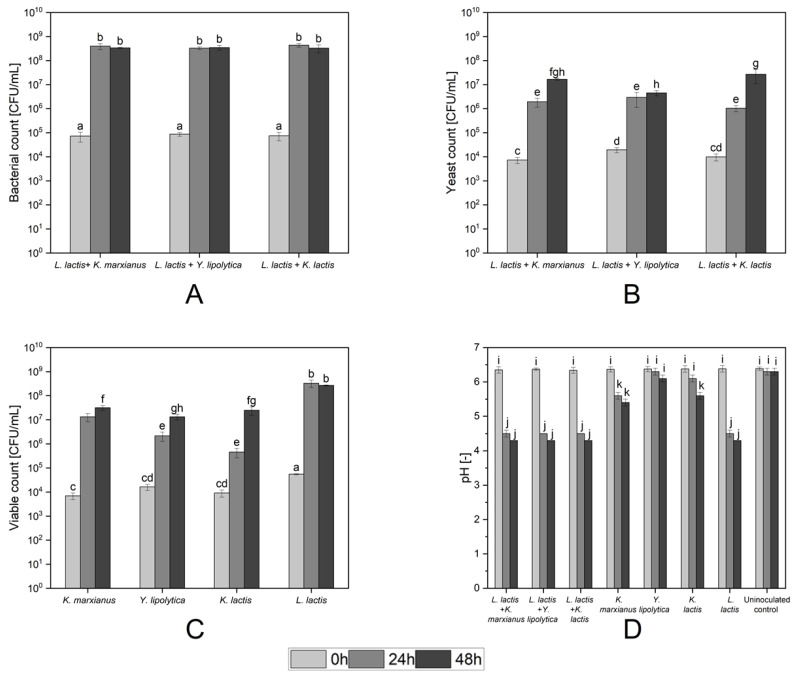
Viable counts and pH-values during fermentation of pea protein suspension by different single and mixed cultures of yeast strains and *L. lactis* LTH 7123. (**A**) Viable cell counts (CFU/mL) of the *L. lactis* LTH 7123 in the mixed strain fermentation. (**B**) Viable counts (CFU/mL) of the yeast strains in the mixed-strain fermentation. (**C**) Viable counts (CFU/mL) of the individual strains during single-strain fermentation. (**D**) Change of pH-values of pea protein throughout 48 h of single-strain and mixed-strain fermentation. Values with the same letters are not significantly different (*p* ≥ 0.05).

The three yeasts strains *K. marxianus* LTH 6039, *K. lactis* LTH 7165, and *Y. lipolytica* LTH 6056 were inoculated as single strains or together with *L. lactis* LTH 7123 for the fermentation of pea protein for 48 h. In addition to the viable counts of each strain, the pH-value of the pea protein suspension was also determined. The lactic acid and ethanol concentrations in the pea protein suspension were determined after 48 h (end of the fermentation). The viable counts of *L. lactis* LTH 7123 and of the yeast strains in the mixed and single-strain fermentations as well as the pH-values of the pea protein suspensions are depicted in Figure 3. The lactic acid and ethanol concentrations of the different fermentation products are listed in Table 3.

The three yeasts were able to grow well as single cultures in the pea protein matrix and reached viable counts ranging from 1.3 to 3.2 × 10^7^ CFU/mL after 48 h (Figure 3C). The highest and fastest growth was determined for *K. marxianus* LTH 6039, reaching 1.3 × 10^7^ CFU/mL after 24 h, followed by *Y. lipolytica* LTH 6056 (2.2 × 10^6^ CFU/mL) and *K. lactis* LTH 7165 (4.6 × 10^5^ CFU/mL). No significant difference (*p* ≥ 0.05) was observed for the viable counts of the yeast strains at the end of the mixed-strain fermentation, compared to the viable count they achieved in the single-strain fermentation.

The pH-value of the pea protein suspension inoculated with the *Kluyveromyces* strains dropped to 5.5 ± 0.2 after 48 h of fermentation (Figure 3D). In the case of *Y. lipolytica* LTH 6056, no significant pH difference was measured (*p* ≥ 0.05). There were no significant differences (*p* ≥ 0.05) observed in the viable counts of *L. lactis* LTH 7123 or the pH-values of the fermentation mixtures after 24 and 48 h of fermentation, whether in single-strain culture or in combination with a yeast strain (Figure 3A,D). In both cases, *L. lactis* LTH 7123 achieved a three-log increase in viable counts already after 24 h accompanied by a decrease in pH-value to 4.5 after 24 h and 4.3 after 48 h.

No differences in the final concentration of L-lactic acid were observed in the presence of yeasts compared to *L. lactis* LTH 7123 alone. The ethanol concentration however was significantly lower (*p* < 0.05) in the mixed-strain fermentation with *L. lactis* LTH 7123 in comparison to the single-strain fermentation of both *Kluyveromyces* yeast strains. No noteworthy ethanol production was observed for *Y. lipolytica* LTH 6056.

### 3.4. Free Amino Acids

**Figure 4:**
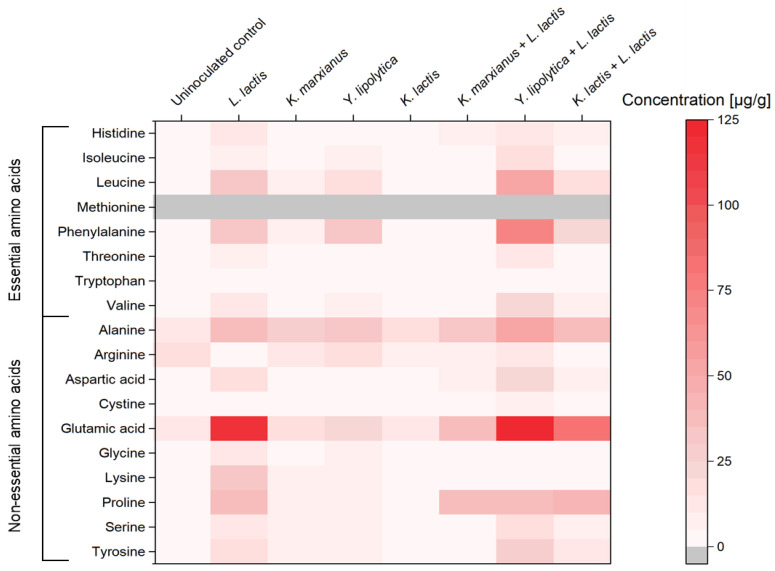
Heatmap of the concentration of essential and non-essential free amino acids in fermented samples. Concentration is given in µg amino acid per one gram of sample. Gray color indicates a concentration below the detection limit of 0.2 µg/g.

The spectrum of free amino acids within the final fermentation products was determined. A specific emphasis was given on monitoring the release of free essential amino acids into the matrix during the fermentation process. In Figure 4, a heatmap of the free amino acids in the fermented samples is displayed, providing a visual representation of the concentration of each amino acid.

The highest levels of total free amino acids were identified in the mixed-strain fermentation sample of *Y. lipolytica* LTH 6056 and *L. lactis* LTH 7123, reaching 496 µg of free amino acids per gram of sample (Figure 4). This was followed by the single strain fermentation product of *L. lactis* LTH 7123, which contained 396 µg/g of total free amino acids. Notably, in both cases, the glutamic acid content increased by tenfold compared to the unfermented control sample, establishing it as the most abundant amino acid in these fermentation products. Arginine was the only amino acid to be depleted in all fermented samples, except in the single-strain fermentation products of *Y. lipolytica* LTH 6056, where it remained unchanged.

The concentration of all essential amino acids, except methionine, in the fermentation product of the combined *L. lactis* LTH 7123 and *Y. lipolytica* LTH 6056 was significantly higher (*p* < 0.05) compared to the control. Specifically, the concentrations of the essential amino acids—histidine, isoleucine, leucine, and phenylalanine—were 40-fold higher in this fermentation product compared to the control. However, the concentration of valine only doubled in comparison. Methionine was not detected in any of the samples.

### 3.5. Aroma Profile and Sensory Characterization of Fermentation Products

**Figure 5:**
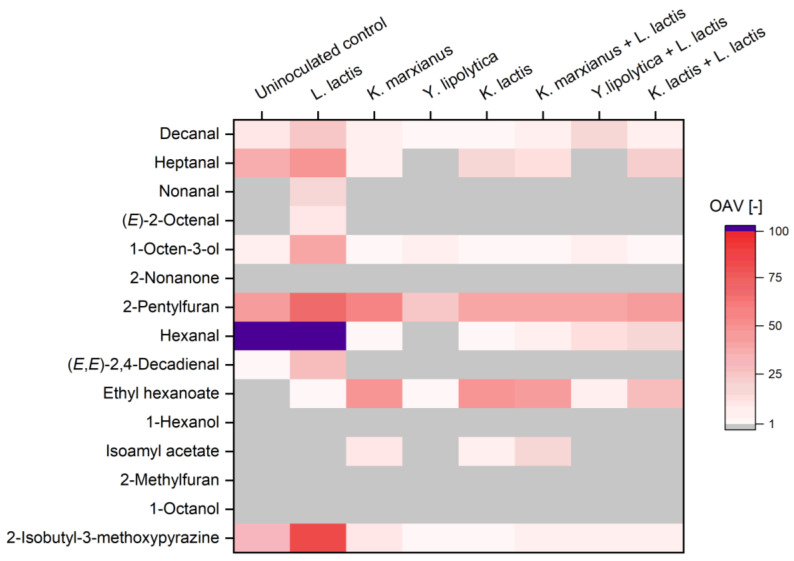
Heatmap of odor activity values (OAV) of volatile aromatic compounds in fermented pea protein suspensions. Gray color indicates a value below OAV of 1 and is considered unperceivable.

The presence and amount of 15 aroma compounds in the various fermentation products were investigated by means of HS-SPME-GC-MS analysis. In Table 4 the concentrations of all compounds in the different samples are given. The odor activity value (OAV) of each single compound in all samples is illustrated as a heatmap in Figure 5. These values express the relative contribution of each compound to the overall aroma. OAV values below 1 are considered unperceivable.

As shown in Table 4, the highest concentration of hexanal (925 µg/L), an important contributor to green hay-like odor of pea, was measured in the fermentation product of *L. lactis* LTH 7123 and was significantly higher (*p* < 0.05) than the concentration of this compound in the uninoculated control sample (616 µg/L). In both, the control and the sample fermented by *L. lactis* LTH 7123, hexanal had the highest OAV, strongly influencing the overall aroma. Other compounds, typically contributing to the green and beany notes of pea, including heptanal, 2-pentylfuran, (*E*,*E*)-2,4-decadienal, decanal, (*E*)-2-octenal, 1-octen-3-ol, and 2-isobutyl-3-methoxypyrazine were also found in higher levels in this fermentation product. To investigate the possible influence of the pH-value on the concentration of volatiles in fermented pea protein samples, an additional control was examined: an uninoculated pea protein suspension adjusted to a pH-value of 4.3, similar to the values determined at the end of the fermentation with *L. lactis* LTH 7123 (Section 2.9). The concentration of each compound present in this control sample is detailed in the Supplementary Materials. Hexanal was found in significantly higher concentrations (*p* < 0.05) in this acidified control compared to the uninoculated non-acidified control. However, its content was notably lower than in the fermented sample produced by *L. lactis* LTH 7123 as a single culture, measuring only 667 µg/L.

Fermentation with each of the three yeast cultures significantly reduced the hexanal content in the pea protein mixture (*p* < 0.05), with the fermentation product of *Y. lipolytica* LTH 6056 achieving the largest reduction to 1.9 µg/L, which is below the sensory odor threshold of this compound (5 µg/L) [41]. Also, the concentrations of the aldehydes heptanal and decanal as well as 2-pentylfuran and 1-octanol were highly reduced by *Y. lipolytica* LTH 6056 compared to the other samples. In samples of pea protein suspension fermented by both a yeast strain and *L. lactis* LTH 7123, a significant reduction in heptanal and hexanal concentrations was observed for all three products. Heptanal concentration was especially low after fermentation with *Y. lipolytica* LTH 6056 in both single and mixed strain fermentation.

Ethyl hexanoate with its fruity odor was detected in high amounts in the samples fermented by one of the two *Kluyveromyces* strains, either single or together with *L. lactis* LTH 7123. The concentration of isoamyl acetate, which is a key odorant of banana, reached high levels in the fermentation products of *K. marxianus* LTH 6039 with 158 µg/L (OAV = 9) alone and 316 µg/L together with *L. lactis* (OAV = 19). This compound was also found in the single-strain fermentation product of *K. lactis* LTH 7165 with 66.1 µg/L (OAV = 4), but not in a notable amount in the yeast mixed-strain fermentation product with *L. lactis* LTH 7123. The concentration of 1-hexanol, however, significantly higher in all fermented products than in the control sample, did not exceed its odor threshold in any of the tested samples. Fermentation with *L. lactis* LTH 7123 as a single strain or in combination, led to the highest concentrations of this compound, with over 800 µg/L in all fermentation products involving this strain. Other compounds with an OAV value below one, and therefore not notably contributing to the overall aroma in all the samples, were 2-nonanone, 2-methylfuran, and 1-octanol.

For a better understanding of the influence of fermentation with the different starter cultures on the aroma profile of the pea protein suspension, a blind and randomized sensory evaluation with five participants (*n* = 5) was conducted. The odor of the different samples was then described and is presented in Table 5.

In general, samples fermented with *Kluyveromyces* strains were described as fruity and fermented, while all other samples were described distinctly as cereal-like, beany and green. Even though, the fermented product of *Y. lipolytica* LTH 6056 and *L. lactis* LTH 7123 was also described as slightly cereal-like, a yeasty note was also perceived.

Overall, the sensory descriptions showed good accordance with the data obtained from the GC-MS analysis and can explain the observed shift in the overall flavor. Other important aroma compounds that were not analyzed in the current study could explain small discrepancies between the results.

## 4. Discussion

Plant-based alternatives to meat and dairy products have recently grown in popularity [4]. As a result, efforts to find adequate plant protein sources to replace animal-based proteins, such as pea protein, have been expanded. Due to technological and sensory issues, as well as food safety concerns the use of plant-based proteins introduced new challenges in the development of new products [7,16,45]. Fermentation is one approach to addressing these problems and producing safer and more appealing products [30,46]. Fermentation experiments with pea protein suspensions were performed and evaluated using single and mixed starter cultures of homofermentative LAB and yeasts. The ability of LAB and yeasts to grow in the pea protein matrix, as well as their effect on various characteristics of the final product, were the primary goal of this study.

Homofermentative LAB strains were assessed for their capacity to acidify the pea protein matrix, aiming to ensure microbiological stability and to prolong shelf-life. The rapid acidification test outlined in this study offers a straightforward method applicable for screening numerous strains not only in pea protein but also in other plant-based proteins. Previous acidification tests applying dyes like carboxyfluorescein were employed in screening acidifying strains in pea protein [27]. Additionally, the analysis of four microbial strains demonstrated a correlation between rapid acidification of the pea protein matrix and accelerated growth, allowing predictive outcomes in the search for suitable starter cultures. Although most strains were capable of acidifying the matrix, fermentation with *L. lactis* LTH 7123 yielded the most significant pH-value reduction in the shortest time frame, alongside the highest cell counts among the tested strains. Engels et al. 2022 also reported that *Lactococcus* strains reached the lowest pH-values in the fermentation of pea protein more than any other strain of LAB genera [27]. A moderate decrease in pH was observed during single strain fermentation with each of the *Kluyveromyces* strains, a phenomenon, which was formerly described [28]. This drop might have been induced by CO_2_ gas production, which, upon dissolving, acidified the matrix, or by the generation of other organic acids by the yeasts [47].

The concentration of free amino acid found in fermentation products of *L. lactis* LTH 7123 confirmed a strong proteolytic activity, which was enhanced by the presence of *Y. lipolytica* LTH 6056. Release of amino acids into the pea protein matrix can improve their bioavailability and elevate the nutritional value of the protein [48]. Furthermore, the substantial presence of free glutamic acid in the fermented pea protein has the potential to enrich the umami flavor, commonly associated with cheese and meat products [49]. *L. lactis* strain LTH 7123 was found to have several cell-envelope peptidases (PrtP), which attribute to its high proteolytic capabilities [50]. Also, *Y. lipolytica* is known for its strong proteolytic system, consisting of extracellular proteases, which are typically synthesized at the end of its logarithmic growth phase in protein-rich environments [51].

The reduction of hexanal concentration by *Y. lipolytica* LTH 6056, alongside an increase in its degradation product hexanol in the fermented pea protein suspension, was also observed during the fermentation of soybean residue by other strains of this yeast [22,52]. This phenomenon stems from the robust lipolytic and β-oxidation activities inherent to *Y. lipolytica* [52]. In contrast, fermentation solely with *L. lactis* LTH 7123 led to an increase in hexanal and hexanol levels, along with the presence of other undesirable flavor compounds. It was shown that an acidic pH (below 4.5) can facilitate the release of bound hexanal from soy proteins, enabling its degradation by LAB [53]. However, it was shown here that the acidic pH-value alone could not fully explain the considerable increase in hexanal by *L. lactis* LTH 7123, implying potential hexanal production by this strain itself. Co-fermentation with a yeast strain, however, efficiently reduces the hexanal in fermented pea protein.

Such a reduction in the hexanal content together with an increase in fruity odors was observed for the two *Kluyveromyces* strains. Ester compounds such as ethyl hexanoate and isoamyl acetate, with their fruity and sweet notes, are generated either by yeast lipid metabolism or as a result of the reaction between higher alcohols and acetyl Co-A [54,55]. While the presence of esters can add more complexity to the overall aroma of pea-based products, it might be undesirable when the final product is be a plant-based meat or dairy alternative.

Although *Y. lipolytica*, a yeast commonly found in fermented foods like cheese and dry sausage, has not been frequently used as a starter culture in food fermentations [56], it serves as a widely utilized biotechnological tool for producing a diverse range of products [57,58]. Notably, applications of this yeast species have received the “Generally Regarded as Safe” (GRAS) status from the American Food and Drug Administration [59]. Furthermore, the European Food Safety Authority (EFSA) has classified *Y. lipolytica* inactivated biomass as a safe novel food with a QPS status [60,61]. Thus, a post-fermentation heat inactivation is necessary when employing this yeast as a starter culture for food production.

## 5. Conclusions

The combination of *L. lactis* LTH 7123 with the yeast strain *Y. lipolytica* LTH 6056 led to fast acidification of the matrix coupled by the growth of both strains without the production of ethanol or the fruity odor associated with other yeast strains. The co-fermentation by both strains enhanced the nutritional value of the pea protein and created a milder sensory profile, which can be used as an improved starting material for the manufacture of plant-based food alternatives. *Y. lipolytica* is a promising food starter culture owing to its robust proteolytic and lipolytic capabilities. However, additional research is necessary to further investigate its functions in food fermentation and in its ability to degrade off-flavors associated with plant-based proteins Nonetheless, until a full authorization to include viable *Y. lipolytica* cells in the final product is obtained, the use of this yeast as a starter culture is limited and must include an inactivation step.

## Figures and Tables

**Table 1:** Microbial strains used in this study.

Strain	Alternative Strain Designation
Bacteria	
*Latilactobacillus curvatus* LTH 955^T^	DSM 20019, ATCC 25601
*Lacticaseibacillus rhamnosus* LTH 1409^T^	DSM 20021, ATCC 7469
*Pediococcus acidilactici* LTH 1412^T^	DSM 20284
*Latilactobacillus sakei* subsp. *sakei* LTH 3745^T^	DSM 20017, ATCC 15521
*Lactiplantibacillus plantarum* LTH 6724	
*Pediococcus pentosaceus* LTH 6727	
*Lactococcus cremoris* LTH 7122	
*Lactococcus lactis* subsp. *lactis* LTH 7123	
*Streptococcus thermophilus* LTH 7138	
*Lactobacillus delbrueckii* subsp. *bulgaricus* LTH 7139	
*Lactococcus lactis* subsp. *lactis* LTH 7163	DSM 20729, ATCC 11454
Yeasts	
*Kluyveromyces marxianus* LTH 6039	DSM 70343
*Yarrowia lipolytica* LTH 6056	
*Kluyveromyces lactis* LTH 7165	DSM 70799, ATCC 8585

T = Type strain.

**Table 2:** Analyzed aroma compounds along with their odor threshold values and odor description (including references).

Compound	Odor Description ^1^	Odor Threshold in Water ^2^ [µg/L]
Decanal	green	0.10
Heptanal	floral, green	3.00
Nonanal	citrus-like	1.00
(*E*)-2-Octenal	green	3.00
1-Octen-3-ol	mushroom-like	1.00
2-Nonanone	grass, fruity, floral [34]	200.00
2-Pentylfuran	beany	6.00
Hexanal	grassy, green	5.00
(*E*,*E*)-2,4-Decadienal	green	0.07
Ethyl hexanoate	fruity	1.00
1-Hexanol	floral, green, perfume-like	2500.00
Isoamyl acetate	sweetish, banana-like [35]	17.00 [36]
2-Methylfuran	cocoa-like, nutty [37]	90.00 [38]
1-Octanol	artificial	130.00
2-Isobutyl-3-methoxypyrazine	bell pepper-like, spicy	0.01 [39]

^1^ Descriptors are taken from Nedele et al., 2021 if no other reference is given [40]. ^2^ Values are taken from Leffingwell and associates data bank 2023 [41] if no other reference is given.

**Table 3:** L-Lactic acid and ethanol contents in fermentation products of *L. lactis* LTH 7123 and yeast strains.

Strains	L-Lactic Acid [g/L]	Ethanol [g/L]
*L. lactis* LTH 7123 + *K. marxianus* LTH 6039	2.6 ± 0.1 ^a^	2.2 ± 0.1 ^d^
*L. lactis* LTH 7123 + *Y. lipolytica* LTH 6056	2.6 ± 0.4 ^a^	0.3 ± 0.1
*L. lactis* LTH 7123 + *K. lactis* LTH 7165	2.9 ± 0.2 ^a^	1.5 ± 0.1 ^d^
*K. marxianus* LTH 6039	0.1 ± 0.0 ^b^	4.3 ± 0.6 ^c^
*Y. lipolytica* LTH 6056	0.1 ± 0.0 ^b^	< 0.001
*K. lactis* LTH 7165	0.1 ± 0.0 ^b^	4.2 ± 1.1 ^c^
*L. lactis* LTH 7123	2.4 ± 0.5 ^a^	<0.001
Uninoculated control	0.1 ± 0.0 ^b^	<0.001

Values with the same letters are not significantly different (*p* ≥ 0.05).

**Table 4:** Concentration of aroma compounds in fermented pea protein samples (µg/L).

Compound	Uninoculated Control	*L.* *lactis*	*K.* *marxianus*	*Y.* *lipolytica*	*K.* *lactis*	*K. marxianus* + *L. lactis*	*Y. lipolytica* + *L. lactis*	*K. lactis* + *L. lactis*
Decanal	1.0 ± 0.4	2.6 ± 0.2	0.3 ± 0.1	0.1 ± 0.0	0.2 ± 0.0	0.3 ± 0.0	1.5 ± 0.5	0.7 ± 0.2
Heptanal	110.6 ± 7.0	151.0 ± 6.8	25.7 ± 7.7	0.4 ± 0.1	59.7 ± 7.3	38.0 ± 3.5	1.0 ± 0.2	66.0 ± 5.6
Nonanal	0.6 ± 0.1	17.7 ± 1.9	0.1 ± 0.0	0.0 ± 0.0	0.1 ± 0.0	0.1 ± 0.0	0.2 ± 0.1	0.1 ± 0.0
(*E*)-2-Octenal	1.0 ± 0.2	27.3 ± 2.8	1.1 ± 0.6	0.1 ± 0.0	0.0 ± 0.0	1.2 ± 0.0	0.2 ± 0.1	0.0 ± 0.0
1-Octen-3-ol	8.1 ± 0.6	38.6 ± 1.9	1.0 ± 0.4	3.7 ± 0.4	0.6 ± 0.1	2.0 ± 0.3	8.0 ± 1.1	2.0 ± 0.3
2-Nonanone	3.4 ± 0.2	8.4 ± 0.7	0.5 ± 0.2	0.2 ± 0.1	0.3 ± 0.0	0.3 ± 0.1	0.3 ± 0.1	0.5 ± 0.2
2-Pentylfuran	283.7 ± 54.8	396.3 ± 23.7	329.9 ± 44.8	157.1 ± 23.4	238.6 ± 17.2	245.5 ± 14.2	237.3 ± 64.4	253.8 ± 29.0
Hexanal	615.9 ± 7.1	924.9 ± 13.8	5.8 ± 1.0	1.9 ± 0.4	5.7 ± 0.8	17.1 ± 4.4	63.8 ± 9.6	89.4 ± 12.4
(*E*,*E*)-2,4-Decadienal	0.0 ± 0.0	1.9 ± 0.7	0.0 ± 0.0	0.0 ± 0.0	0.0 ± 0.0	0.0 ± 0.0	0.0 ± 0.0	0.0 ± 0.0
Ethyl hexanoate	0.4 ± 0.01	0.8 ± 0.1	47.8 ± 3.8	1.2 ± 0.3	47.9 ± 3.5	41.8 ± 3.3	2.6 ± 0.5	27.0 ± 2.9
1-Hexanol	40.0 ± 0.8	1073.1 ± 52.0	461.2 ± 40.6	142.8 ± 14.9	485.9 ± 15.0	1058.9 ± 78.3	829.1 ± 119.3	1161.4 ± 107.4
Isoamyl acetate	0.0 ± 0.0	0.1 ± 0.0	158.3 ± 15.7	0.0 ± 0.0	66.1 ± 1.5	316.4 ± 14.2	0.2 ± 0.1	0.6 ± 0.4
2-Methylfuran	3.1 ± 0.8	5.5 ± 0.7	1.4 ± 0.4	4.5 ± 1.2	1.9 ± 0.2	1.7 ± 0.3	5.5 ± 1.1	2.0 ± 0.3
1-Octanol	1.9 ± 0.3	21.3 ± 1.8	12.2 ± 1.0	0.0 ± 0.0	2.1 ± 0.0	12.0 ± 2.7	0.8 ± 0.3	8.3 ± 0.9
2-Isobutyl-3-methoxypyrazine	0.4 ± 0.2	1.0 ± 0.1	0.1 ± 0.0	0.0 ± 0.0	0.0 ± 0.0	0.0 ± 0.0	0.0 ± 0.0	0.1 ± 0.0

**Table 5:** Sensory evaluation of fermented pea protein products by different starter cultures.

Strain	Descriptors
*L. lactis* LTH 7123 + *K. marxianus* LTH 6039	fermented, alcoholic, sourish
*L. lactis* LTH 7123 + *Y. lipolytica* LTH 6056	yeasty, slightly cereal-like
*L. lactis* LTH 7123 + *K. lactis* LTH 7165	fermented, malty, milky
*K. marxianus* LTH 6039	fruity, malty, dough-like, sweetish
*Y. lipolytica* LTH 6056	green, cereal-like, beany
*K. lactis* LTH 7165	fruity, grape pomace-like, alcoholic
*L. lactis* LTH 7123	cereal-like, soy-like, beany, weakly sourish
Uninoculated control	cereal-like, green, soy drink-like, beany

## Data Availability

The data presented in this study are available on request from the corresponding author.

## Supplementary Materials

The following supporting information can be downloaded at: https://www.mdpi.com/article/10.3390/foods13040588/s1, Table S1: Concentration of aroma compounds in acidified pea protein suspension.
