# Supplementary material for: Rapid Acidification and Off-Flavor Reduction of Pea Protein by Fermentation with Lactic Acid Bacteria and Yeasts
Source: Foods. 2024 Feb 15;13(4):588. doi: 10.3390/foods13040588 (PMC10888418; doi:10.3390/foods13040588)
Supplement: Supplementary file 1 [file foods-13-00588-s001.zip › foods-2849575-supplementary.pdf]

Supplementary Material

Table S1. Concentration (µg /L) of aroma compounds in acidified pea protein suspension with a final pH-value of 4.3.

Compound	Acidified Control Sample
Decanal	0.8 ± 0.1
Heptanal	141.4 ± 4.5
Nonanal	1.5 ± 0.1
(*E*)-2-Octenal	2.6 ± 0.3
1-Octen-3-ol	9.6 ± 0.3
2-Nonanone	4.8 ± 0.1
2-Pentylfuran	321.1 ± 15.5
Hexanal	666.6 ± 22.2
(*E*,*E*)-2,4-Decadienal	0.3 ± 0.0
Ethyl hexanoate	0.3 ± 0.0
1-Hexanol	39.5 ± 1.1
Isoamyl acetate	0.0 ± 0.0
2-Methylfuran	2.4 ± 0.2
1-Octanol	2.8 ± 0.5
2-Isobutyl-3-methoxypyrazine	0.4 ± 0.2
